# The Impact of a Severe Drought on Dust Lifting in California’s Owens Lake Area

**DOI:** 10.1038/s41598-017-01829-7

**Published:** 2017-05-11

**Authors:** Cauê S. Borlina, Nilton O. Rennó

**Affiliations:** 1 Massachussets Institute of Technology, Department of Earth, Atmospheric and Planetary Sciences, Cambridge, MA USA; 2 University of Michigan, Department of Climate and Space Sciences and Engineering, Ann Arbor, Michigan USA

## Abstract

Mineral dust aerosols are responsible for some of the largest sources of uncertainties in our current understanding of climate change. Here we show that a severe drought is having a significant impact in one of largest sources of mineral dust aerosols of the U.S., the Owens Lake area in California’s southwest. Measurements of aerosol concentration (PM2.5 particle matter) in the Owens Lake salty playa show that the annual mean concentration of PM2.5 aerosol has been increasing steadily since the beginning of the current drought, with periods of high aerosol concentration increasing from 4 months in 2013 to 9 months in 2015. Interestingly, the PM2.5 aerosol concentration usually increases abruptly from less than ~0.05 mg/m^3^ to ~0.25 mg/m^3^. This occurs when saltation events break salt crusts produced by the efflorescence of brine in the salty playa. The brine is produced by either rainfall or runoff water. Based on this observation, we hypothesize that there is an upper limit of ~0.25 mg/m^3^ in the annual mean PM2.5 aerosols concentration in the Owens Lake basin that might limit the impact of mineral dust aerosols on climate. Indeed, the upper annual limit of ~0.25 mg/m^3^ has been nearly reached during the current drought.

## Introduction

The effects of aerosols on the Earth’s radiation budget are one of the largest sources of uncertainties in our current understanding of climate change^1^. In order to reduce these uncertainties, we have been studying the relationship between aerosols and climate variability in California’s Owens Lake area, on the eastern side of the Sierra Nevada. The Owens Lake dried as a result of the diversion of the Owens River into the Los Angeles Aqueduct in 1913, becoming one of the largest sources of mineral dust aerosols of the US^2–8^. This dust is made up of sediment from the lakebed which includes waste from the Cerro Gordo mine that was active until 1919^9^. The airborne dust is composed of particles from this anthropogenic source, from the natural desert, and from urban areas of the South Coast of California and the San Joaquin basin^10^. As a result, up to 60–95% of the aerosol mass could be from anthropogenic sources^11^.

California’s current drought provides a unique opportunity for studying the effects of climate variability on aerosol concentration in this important dust source region. We have been monitoring weather, soil water content, radiation fluxes, PM2.5 aerosol concentration (this size class was chosen because it has direct implications for respiratory problems^12^), and studying dust lifting processes at our field site in the Owens Lake salty playa since December of 2012^13^. Here we report results of the analysis of PM2.5 aerosol concentration, saltation activity, soil water content, and rainfall from January 2013 to December 2015.

**Figure 1:**
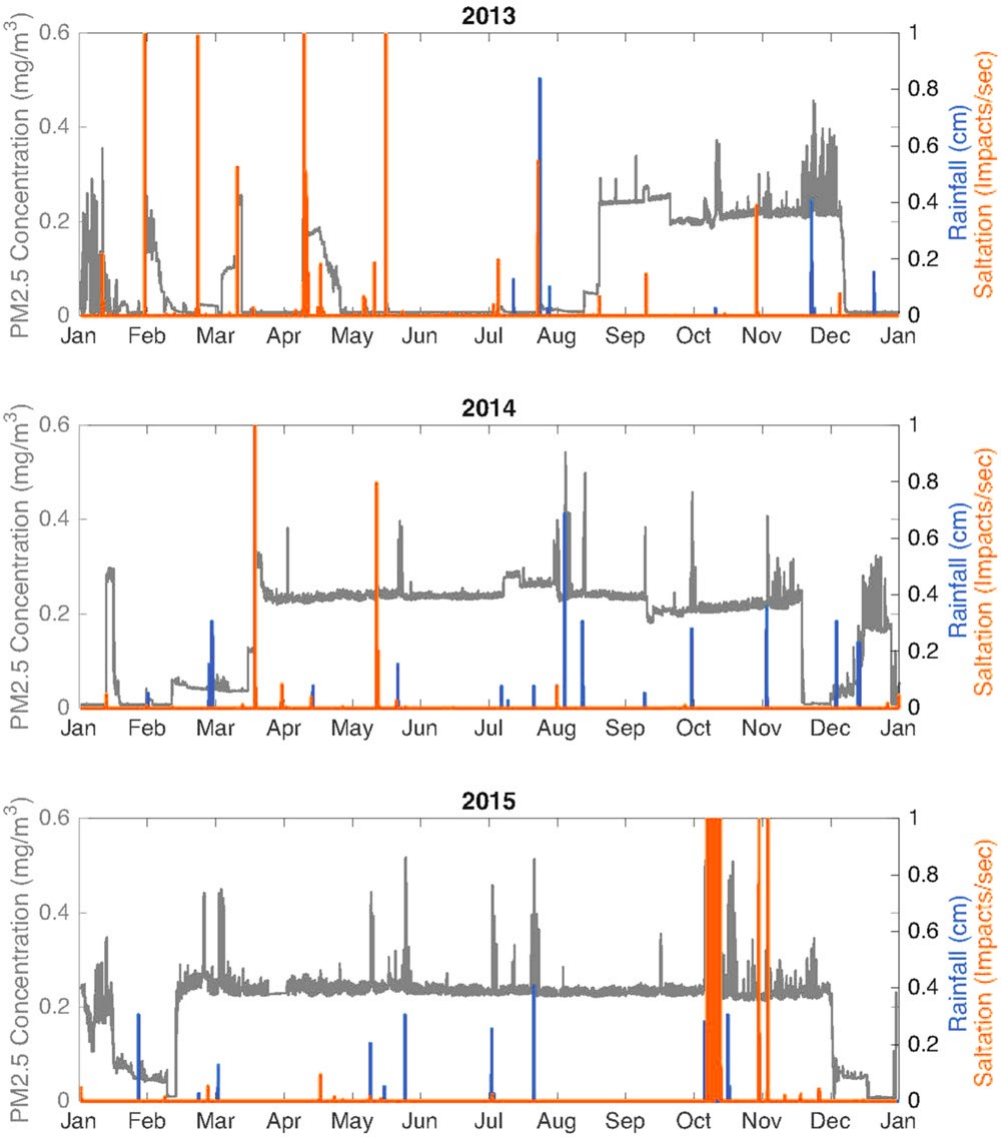
Time series of PM2.5 aerosol concentration, saltation, and precipitation at our field site in the Owens Lake salty playa in 2013, 2014 and 2015. These indicate that saltation events usually produce abrupt and large increases in aerosol concentration.

**Figure 2:**
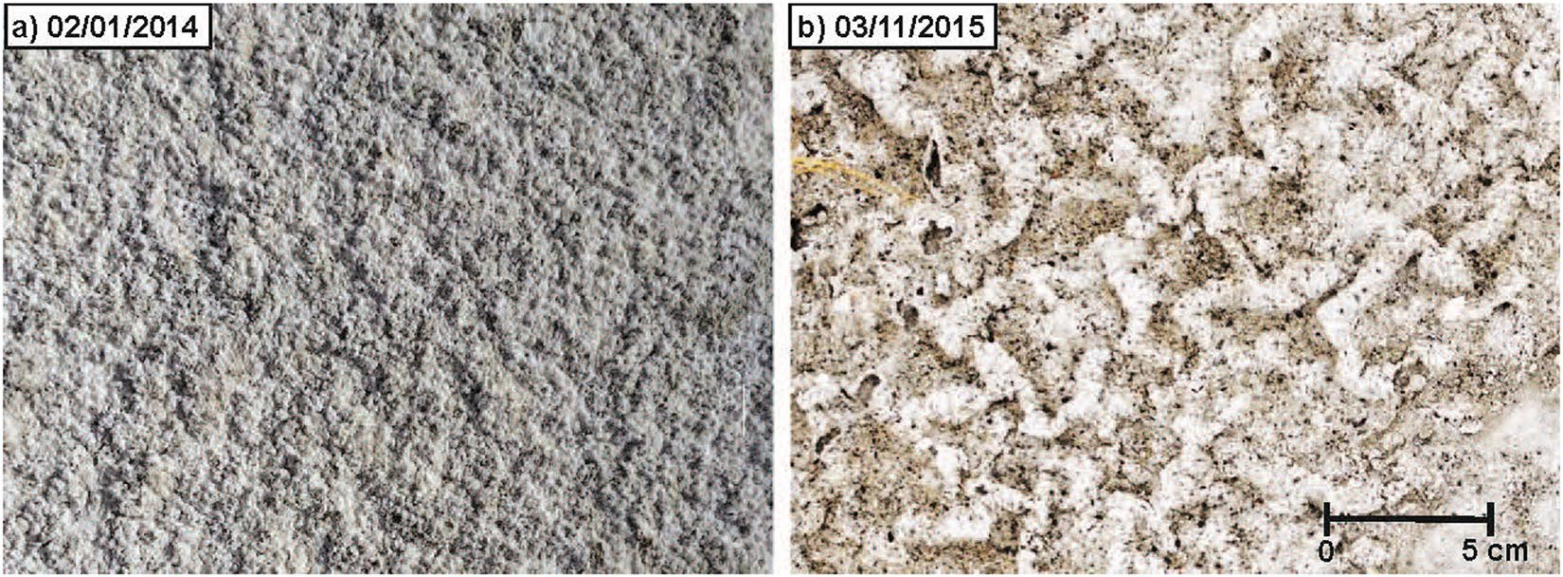
Comparison of surface crusts observed on (**a**) February 1^st^ of 2014 and (**b**) March 11^th^ of 2015. Images were taken before (**a**) and after (**b**) the abrupt increase in aerosol concentration (Fig. 1). The fragmented surface salt crust observed in (**b**) suggests that the breaking of surface salt crusts produces the increase in aerosol concentration.

**Figure 3:**
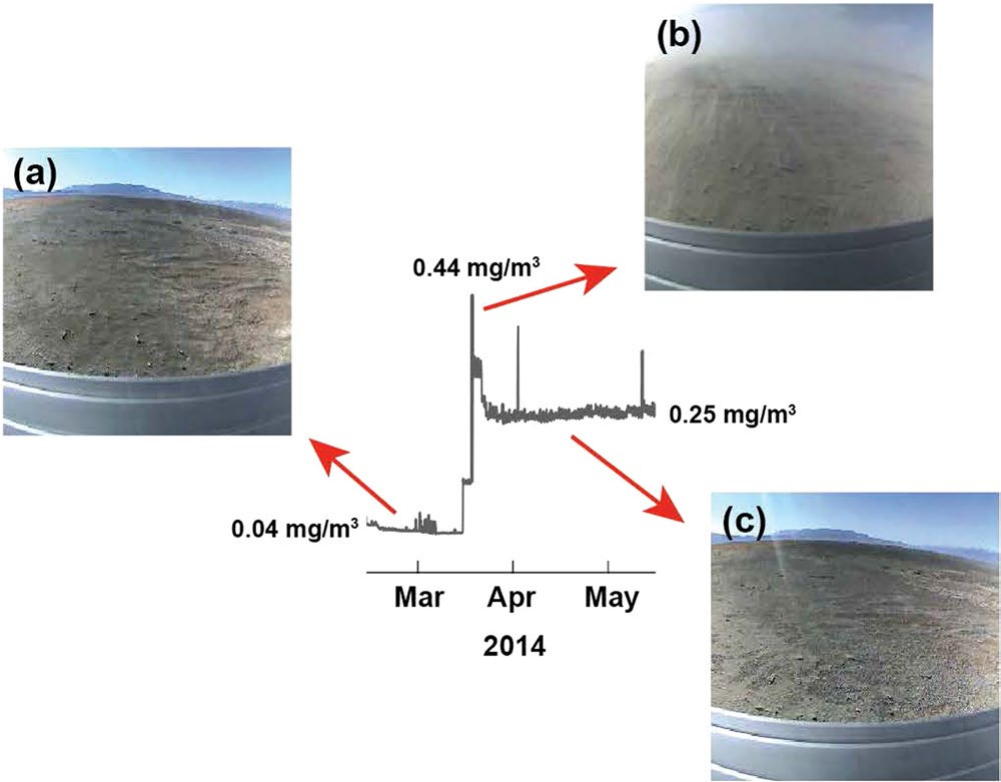
Images taken before (**a**), during (**b**) and after (**c**) a saltation event in 2014 shows that the abrupt increase in the aerosol concentration at the field site was caused by the breaking of surface salt crusts. The data in the center is a snap shot of that presented in Fig. 1. This major dust-lifting event caused an abrupt increase in the PM2.5 aerosol concentration.

Figure 1 shows that the aerosol concentration frequently increases abruptly from a low background value of ~0.05 mg/m^3^ to ~0.25 mg/m^3^, as occurred in mid-August 2013, mid-March 2014 and mid-February 2015. Abrupt decreases in surface irradiance independently confirm the abrupt increases in aerosol concentration (Figs S1–3), but usually the interpretation of irradiance data is complex because of the presence of clouds, the effects of dust deposition on the sensor, and seasonal changes in irradiance. The breaking of new surface salt crusts by intense saltation events causes the abrupt increases in aerosol concentration that we have been observing (Fig. 1). The salt crusts, in turn, form when brine produced by the accumulation of either rainfall or runoff water on the salty playa effloresces, cementing the saline topsoil^1, 2, 4, 13^. Previous studies have shown that salt crusts expand and contract with hydration and dehydration, breaking easily when expanded^1^. Figure 2a indicates that in February 2014 new salt crusts are present in our field site. Figure 2b indicates that by the middle of the second week of March 2014, hydration has forced these salt crusts to expand, making them easily breakable. In mid-March, an intense saltation event breaks the salt crusts and increases the PM2.5 aerosol concentration by an order of magnitude. The breaking of the salt crusts is illustrated in the images taken before (Fig. 3a), during (Fig. 3b), and after a saltation event in mid-March 2014 (Fig. 3c). Similar events occurred in mid-August 2013, and in mid-February 2015. Usually, late in the fall the wetting of the surface by precipitation and runoff inhibits dust lifting, reducing the aerosol concentration to typical low background values^2, 7^.

Measurements of PM2.5 aerosol concentration, saltation, precipitation, near surface atmospheric relative humidity, and soil volumetric water content clearly indicates that the periods of elevated aerosol concentration are triggered by saltation events (Figs S4–6). These events occur when the volumetric soil water content is high (above ~0.8 kg/m^3^). This observation and the fact that the relative humidity of the near surface air is usually lower during the periods of elevated aerosol concentration indicates that salt crusts inhibit evaporation, contributing to increases in soil water content. The fact that the aerosol concentration increases with soil water content is consistent with previous results showing that aerosol concentration increases with the humidity of the topsoil^13^.

Based on the results described above, we hypothesize that the elevated aerosol concentration values observed in 2013, 2014 and 2015 are at least partially the result of nearly continuous breaking of new salt crusts forming on water saturated soil, without the need for precipitation. The fact that these salt crusts saltate easily explains why the periods of elevated aerosol concentration are much longer in 2015 than in 2013.

Aerosol concentration has been increasing significantly with human activity, and current estimates suggest that the global aerosol climate forcing might be as large as a factor of two of the forcing due to greenhouse gases^14^. Regionally, seasonal aerosol climate forcing can be much larger and might reach up to ten times the forcing due to greenhouse gases^15^. Aerosols produce a direct radiative forcing by scattering and absorbing solar and infrared radiation, and cause an indirect radiative forcing by altering cloud processes via increases in cloud droplet number and ice particle concentration. Thus, a better understanding of the interactions between aerosols and climate is necessary for reducing some of the main sources of uncertainty in climate predictions.

An upper limit of ~0.25 mg/m^3^ in the annual mean concentration of PM2.5 aerosols would place an upper bound on the effects of mineral dust aerosols on climate. This limit appears to exist because severe drought increases the periods of high PM2.5 aerosol concentration, but not the value of the mean aerosol concentration during these periods. This is important for the general public and therefore policy makers because the high level of uncertainty in current climate predictions is one the main reasons why their use has lagged in decision making^16^.

## Methods

The data analyzed in this article was collected at Owens Lake by the Aerosols-Climate Interaction project in California’s Owens Lake area^13^. Fine particulate matter (or PM2.5) was measured using a Met-One E-sampler, saltation was measured using a Sensit H11-LIN, soil volumetric water content was measured 30 cm below the surface with a Campbell-Scientific CS616 sensor, and direct reflectance measured using a Campbell-Scientific CNR4. All measurements were conducted at a frequency of 1 Hz and the data was averaged hourly. Rainfall data was collected by the Great Basin Unified Air Pollution Control District with the A-Tower, and reported hourly.

## Electronic material

Supplementary Material
