# Supplementary material for: The Impact of a Severe Drought on Dust Lifting in California’s Owens Lake Area
Source: Sci Rep. 2017 May 11;7:1784. doi: 10.1038/s41598-017-01829-7 (PMC5431890; doi:10.1038/s41598-017-01829-7)
Supplement: Supplementary file 1 — Supplementary Material [file 41598_2017_1829_MOESM1_ESM.pdf]

Supplementary Information

The Impact of a Severe Drought on Dust Lifting in California's Owens Lake Area

Cauê S. Borlina & Nilton O. Rennó

1. Inferring Increases in Aerosol Concentration from Measurements of Irradiance

Decrease in irradiance at our field site at the Owens Lake provides independent confirmation of the increase in PM2.5 aerosol concentration (Figs. S1, S2 and S3). In this case, the irradiance clearly decreased when the PM2.5 aerosol concentration increased (Fig. S1). Unfortunately, usually the interpretation of the irradiance data is complex because of the effects of dust deposition on the irradiance sensor, the possible presence of clouds, and changes in irradiance with season. This complex behavior is reflected on the results presented in Figs. S2 and S3.

2. Impacts of Volumetric Water Content at Owens Lake Playa

Measurements of soil **v**olumetric water content at our field site at the Owens Lake indicates that even as the severity of the drought increased, the soil has remained nearly saturated (volumetric water content $\gtrsim$ 80%) (Figs. S4, S5 and S6). The instrument that we use to measure the soil volumetric water content (a Campbell-Scientific CS616) requires constant recalibration in order to produce reliable absolute values. Since, we have not been recalibrating the sensor because it is installed in our remote field site at the Owens Lake, our data can be used to estimate relative changes in the soil water content, not absolute values. Based on the response of the sensor during periods of intense rainfall, we estimate that soil volumetric water content of about 80% indicates that the soil is saturated.

Based on the timing and duration of soil saturation events, we hypothesize that saturation causes the formation of salt crusts on the surface and that these salt crusts inhibit evaporation. Our data also shows that high aerosol concentration is correlated with high values of soil volumetric water content, as reported previously[1]. Given the local soil chemistry and how ephemeral crusts are generated at Owens Lake playa, we propose that the high PM2.5 aerosol concentration observed during 2013, 2014 and 2015 (and its long lasting periods) could be the result of the breaking of new salt crusts forming on the top of water saturated soil, without the need for rainfall (data obtained from) to form new crusts. An initial rainfall or runoff event is enough to saturate the soil and create a water reservoir that generate easily breakable surface crusts during prolonged drought periods. This mechanism could explain why high PM2.5 aerosol concentration values last most of the year during severe droughts.

3. References

1. Ramanathan, V., Bates, T. S., Hansen, J. E., Jacob, D. J., Kaufman, Y. J., Penner, J. E., ... & Seinfeld, J. H. (2002). National Aerosol-Climate Interactions Program (NACIP), A National Research Imperative. *White Paper*.

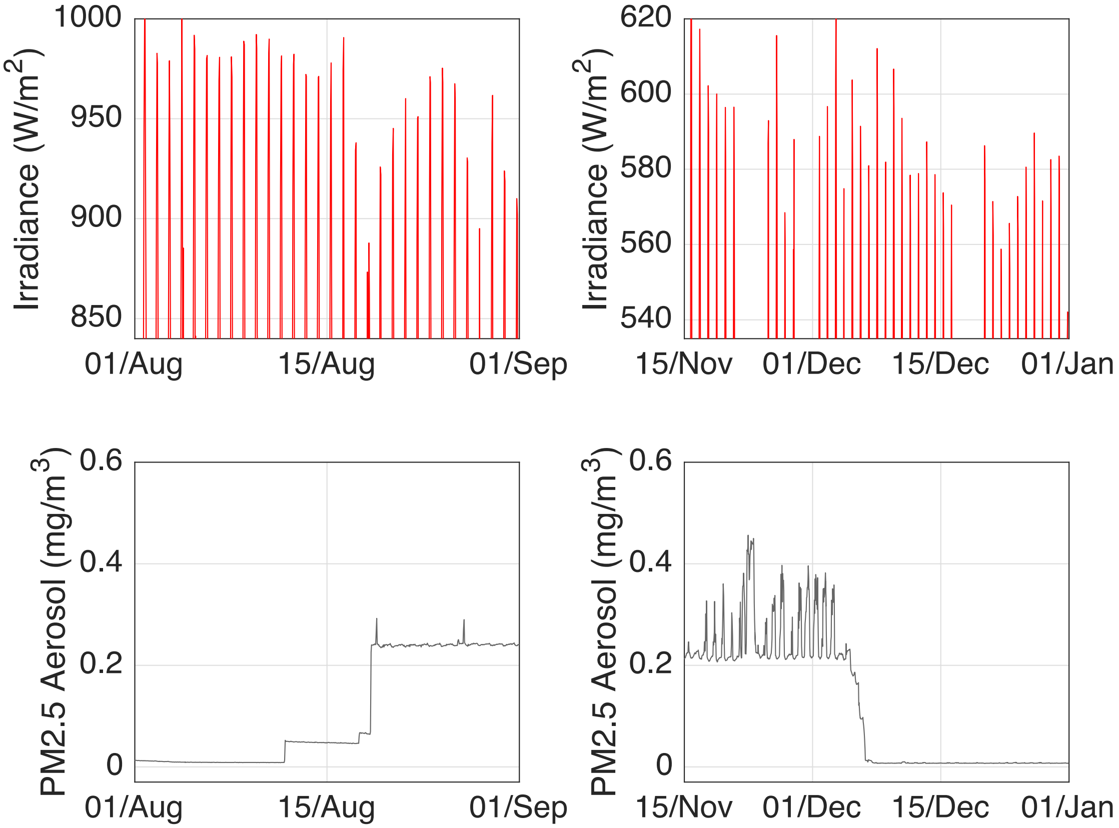

Figure S1. Measurements of irradiance and PM2.5 for the period when the aerosol concentration increases in 2013.

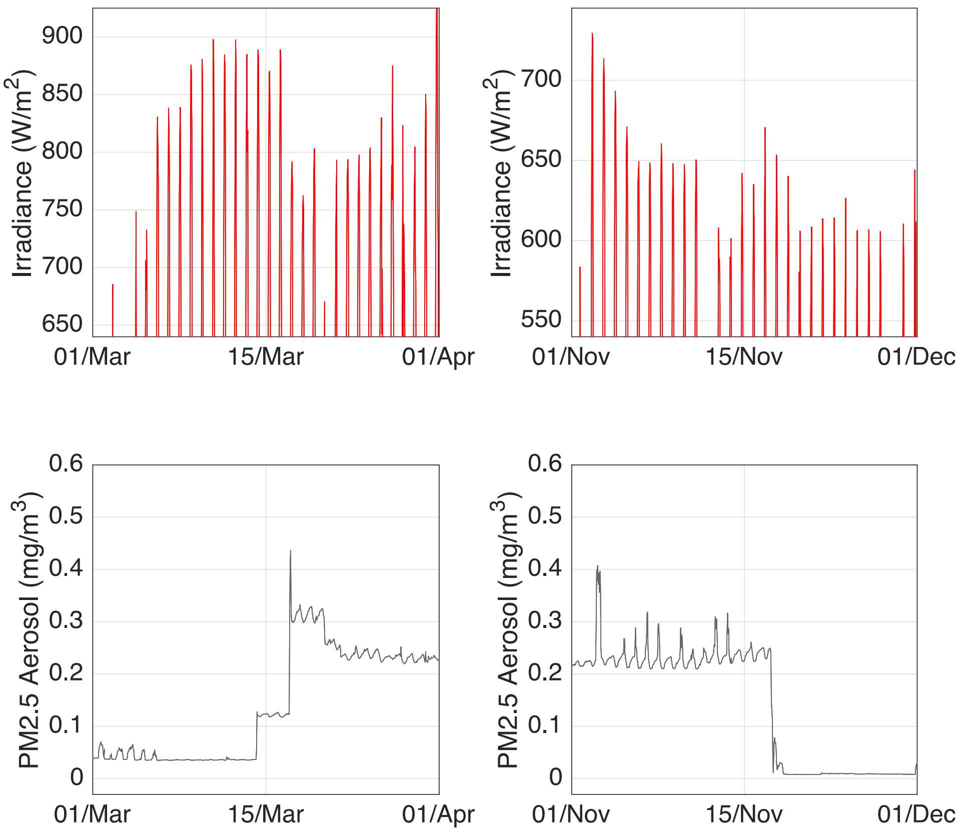

Figure S2. Measurements of irradiance and PM2.5 for the period when the aerosol concentration increases in 2014.

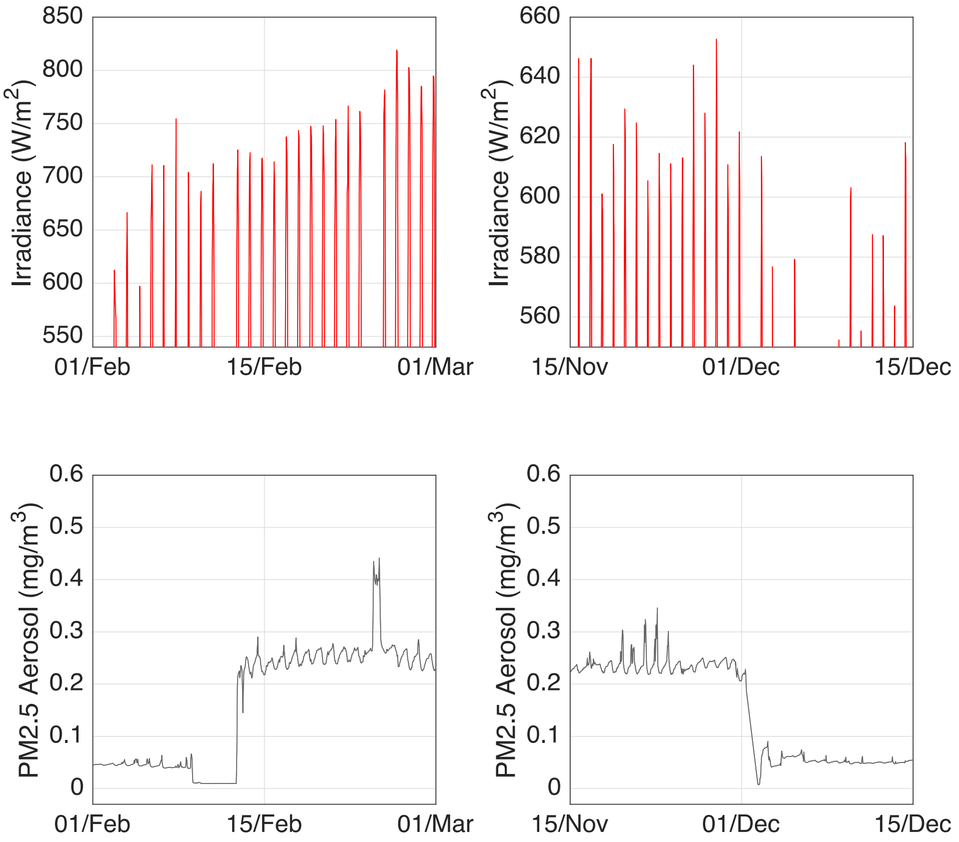

Figure S3. Measurements of irradiance and PM2.5 for the period when the aerosol concentration increases in 2015.

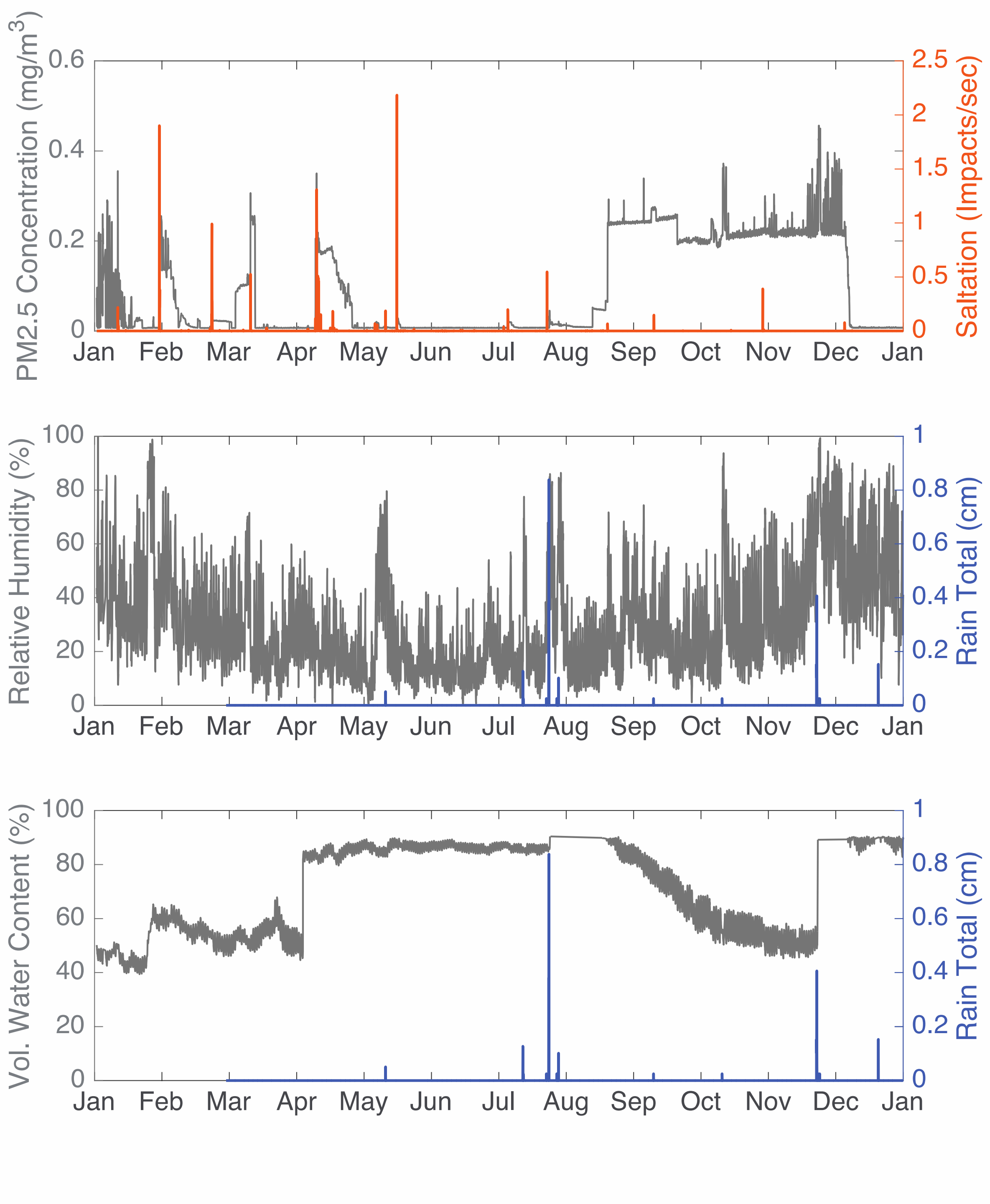

Figure S4. Aerosol concentration, relativity humidity, saltation, rain total, and volumetric water content for the year of 2013.

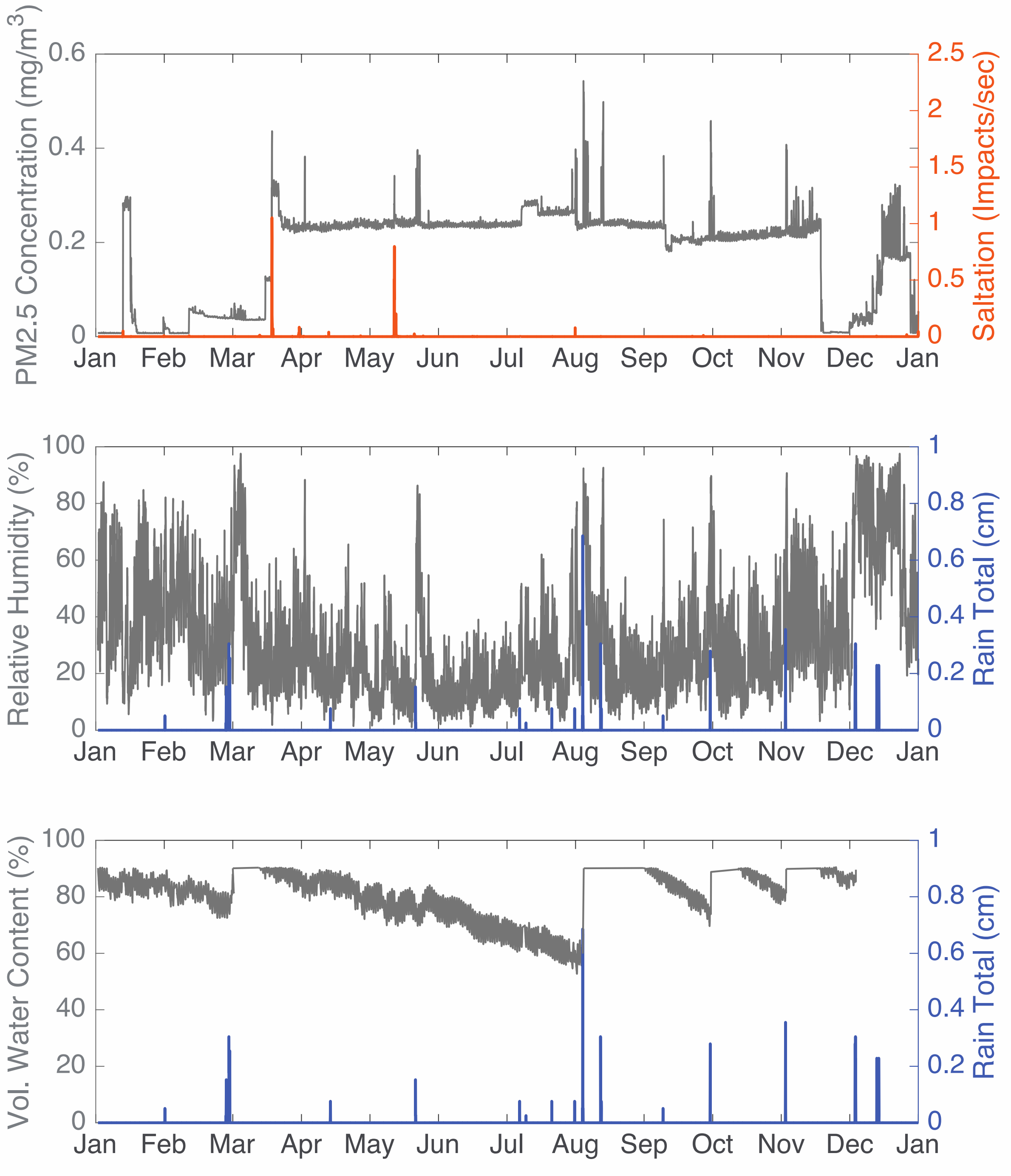

Figure S5. Aerosol concentration, relativity humidity, saltation, rain total, and volumetric water content for the year of 2014.

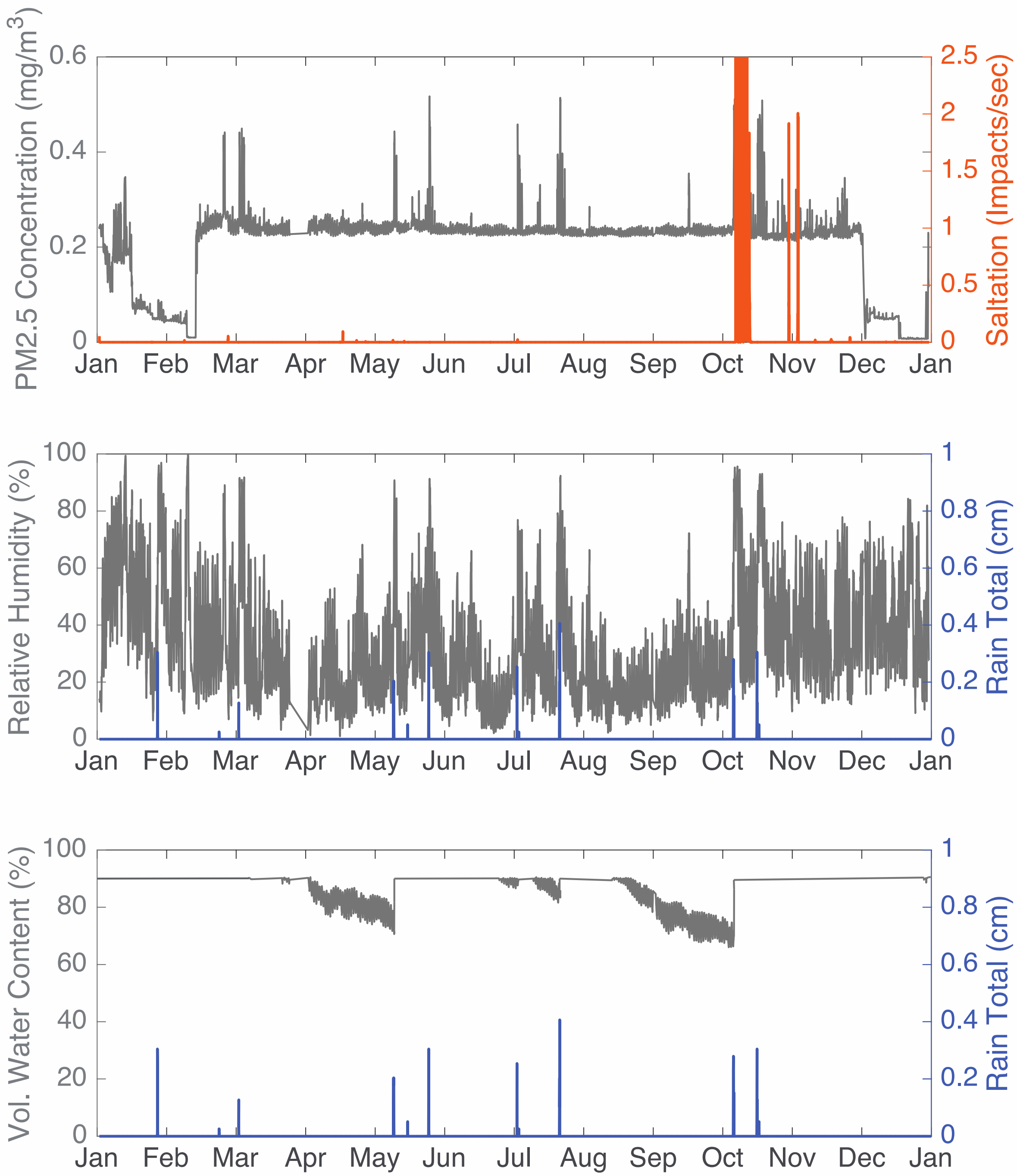

Figure S6. Aerosol concentration, relativity humidity, saltation, rain total, and volumetric water content for the year of 2015.
